# Mechanical ventilation modulates TLR4 and IRAK-3 in a non-infectious, ventilator-induced lung injury model

**DOI:** 10.1186/1465-9921-11-27

**Published:** 2010-03-03

**Authors:** Jesús Villar, Nuria E Cabrera, Milena Casula, Carlos Flores, Francisco Valladares, Lucio Díaz-Flores, Mercedes Muros, Arthur S Slutsky, Robert M Kacmarek

**Affiliations:** 1 CIBER de Enfermedades Respiratorias, Instituto de Salud Carlos III, Spain; 2 Multidisciplinary Organ Dysfunction Evaluation Research Network (MODERN), Research Unit, Hospital Universitario Dr. Negrin, Las Palmas de Gran Canaria, Spain; 3 Keenan Research Center at the Li Ka Shing Knowledge Institute of St. Michael's Hospital, Toronto, Canada; 4 Research Unit, Hospital Universitario N.S. de Candelaria, Tenerife, Spain; 5 Department of Anatomy, Pathology & Histology, University of La Laguna, Tenerife, Spain; 6 Department of Clinical Biochemistry, Hospital Universitario NS de Candelaria, Tenerife, Spain; 7 Interdepartmental Division of Critical Care Medicine, University of Toronto, Toronto, Canada; 8 King Saud University, Riyadh, Saudi Arabia; 9 Department of Respiratory Care, Massachusetts General Hospital, Boston, Massachusetts, USA; 10 Department of Anesthesia, Harvard Medical School, Boston, MA, USA

## Abstract

**Background:**

Previous experimental studies have shown that injurious mechanical ventilation has a direct effect on pulmonary and systemic immune responses. How these responses are propagated or attenuated is a matter of speculation. The goal of this study was to determine the contribution of mechanical ventilation in the regulation of Toll-like receptor (TLR) signaling and interleukin-1 receptor associated kinase-3 (IRAK-3) during experimental ventilator-induced lung injury.

**Methods:**

Prospective, randomized, controlled animal study using male, healthy adults Sprague-Dawley rats weighing 300-350 g. Animals were anesthetized and randomized to spontaneous breathing and to two different mechanical ventilation strategies for 4 hours: high tidal volume (V_T_) (20 ml/kg) and low V_T _(6 ml/kg). Histological evaluation, TLR2, TLR4, *IRAK3 *gene expression, IRAK-3 protein levels, inhibitory kappa B alpha (IκBα), tumor necrosis factor-alpha (*TNF-α*) and interleukin-6 (*IL6*) gene expression in the lungs and TNF-α and IL-6 protein serum concentrations were analyzed.

**Results:**

High V_T _mechanical ventilation for 4 hours was associated with a significant increase of TLR4 but not TLR2, a significant decrease of *IRAK3 *lung gene expression and protein levels, a significant decrease of IκBα, and a higher lung expression and serum concentrations of pro-inflammatory cytokines.

**Conclusions:**

The current study supports an interaction between TLR4 and IRAK-3 signaling pathway for the over-expression and release of pro-inflammatory cytokines during ventilator-induced lung injury. Our study also suggests that injurious mechanical ventilation may elicit an immune response that is similar to that observed during infections.

## Background

Ample evidence from experimental studies suggests that lung overdistension during mechanical ventilation (MV) causes or exacerbates lung injury [1]. Referred to as ventilator-induced lung injury (VILI), this condition may be difficult to diagnose in humans because its appearance may overlap the damage associated with the primary disease for which MV was instituted. Several studies have demonstrated that certain MV strategies lead to induction, synthesis and release of proinflammatory cytokines from the lungs soon after initiation of MV [2-5]. High circulating and tissue levels of proinflammatory cytokines, such as tumor necrosis factor-alpha (TNF-α) and interleukin-6 (IL-6), appear to contribute to the development of a systemic inflammatory response that produces or aggravates lung damage and may lead to multiple organ failure [6]. However, the exact mechanism by which this pro-inflammatory response is initiated, propagated or perpetuated are still not well understood.

Most pulmonary cells express a large repertoire of genes under transcriptional control that are modulated by biomechanical forces [7,8] and bacterial infections [9]. Essential components of the innate immune system are the toll-like receptors (TLRs) [10] which recognize not only microbial products but also degradation products released from damaged tissue providing signals that initiate inflammatory responses [11]. Several different components are involved in TLR signaling, such as IL-1 receptor-associated kinases (IRAK), leading to nuclear translocation of nuclear factor-κB (NF-κB) and ultimately to activation of pro-inflammatory cytokines, such as TNF-α and IL-6 [9,10,12].

Current evidence indicates that IRAK-3 (also known as IRAK-M) is a negative regulator of the TLR pathways and a master regulator of NF-κB and inflammation [13,14]. Several known pathways can lead to NF-κB activation. The classical (canonical) pathway involves the activation of IKKα/β heterodimer, degradation of the inhibitory kappa B alpha (IκBα), and release of p65/p50 from the cytoplasma into the nucleus [15]. The alternative (non-canonical) NF-κB pathway involves NF-κB-inducing kinase-mediated IKKα-dependent cleavage and nuclear translocation of p52/RelB [15]. In both pathways, IRAK-3 selectively inhibits NF-κB activation [13,15]. Since Vaneker et al [16] have recently reported that MV in healthy mice resulted in enhanced *TLR4 *gene expression in lung homogenates, the goal of the present study was to determine the contribution of MV in the regulation of TLR and IRAK-3 signaling in a non-infectious, experimental model of ventilator-induced lung injury.

## Methods

### Animal preparation

The experimental protocol was approved by the Hospital Universitario N.S. de Candelaria Research Committee and the Committee for the Use and Care of Animals, University of La Laguna, Tenerife, Spain, and performed under the European Guidelines for Animal Research. We studied healthy, pathogen-free, male Sprague-Dawley rats (CRIFFA, Barcelona, Spain) weighing 300-350 gm. Animals were anesthetized by intraperitoneal injection of 50 mg/kg body weight ketamine hydrochloride and 2 mg/kg body weight xylazine. Anesthetized animals were randomly allocated into three groups: non-ventilated, ventilated with low tidal volume (V_T_), and ventilated with high V_T_. One group of animals (n = 6) was anesthetized and not ventilated for 4 hours and served as anesthetized, spontaneous breathing controls. In animals assigned to MV, a cervical tracheotomy was performed under general anesthesia using a thin-walled 14-gauge Teflon catheter. After the catheter was secured by a ligature around the trachea, those animals allocated to MV were paralyzed with 1 mg/kg of pancuronium bromide and connected to a time-cycled, volume-limited rodent ventilator (Ugo Basile, Varese, Italy).

### Experimental protocol

Following all surgical procedures, ventilated animals were randomly assigned to either (i) a low V_T _(6 ml/kg) (n = 6) or (ii) a strategy causing ventilator-induced lung injury with a high V_T _(20 ml/kg) (n = 6) on room air and at 0 cmH_2_O of positive end-expiratory pressure (PEEP). In order to minimize the possibility of triggering an inflammatory response by invasive procedures, we were extremely careful to reduce the possibility of contamination by performing our experiments following standard clean surgical procedures and in animals that were monitored non-invasively, after establishing a protocol which provided hemodynamic stability and comparable blood gases between both ventilated groups in invasively monitored healthy animals. In pilot studies, we monitored animals invasively by inserting plastic catheters (Intramedic, Clay Adams, Parsippany, NJ) into the carotid artery for arterial blood sampling and arterial blood pressure monitoring and into the jugular vein for central venous pressure monitoring, and found that the two ventilatory strategies provided hemodynamic stability (mean arterial blood pressure above 70 mmHg and mean central venous pressure above 3 cmH_2_O, respectively, throughout the whole experimental period) and comparable blood gases on room air at the end of 4 hours (PaO_2 _94 ± 4 vs. 89 ± 6 mmHg, PaCO_2 _43 ± 3 vs. 36 ± 4 mmHg, and pH 7.38 ± 0.02 vs. 7.43 ± 0.01, for the low V_T _and high V_T _groups, respectively (n = 5 rats/group). Respiratory rate was set to maintain constant minute ventilation in both groups. Peak inspiratory pressures were continuously monitored. These settings were maintained for 4 hours while supine on a restraining board inclined 20° from the horizontal and anesthetized with ketamine/xylazine and paralyzed with pancuronium bromide. Rectal temperature was monitored and maintained at 36-36.5°C with a heating pad.

### Histological examination

At the end of the 4-hour ventilation period, a midline thoracotomy/laparotomy was performed in all rats and the abdominal vessels were transected. After death, the hearts and lungs were removed *en bloc *from the thorax. Then, the lungs were isolated from the heart, the trachea was cannulated and the right lung was fixed by intratracheal instillation of 3 ml of 10% neutral buffered formalin. After fixation, the lungs were floated in 10% formalin for a week. Lungs were serially sliced from apex to base and specimens were embedded in paraffin, then cut (3 μm thickness), stained with hematoxylin-eosin and examined under light microscopy. Two pathologists (FV, LDF), blinded to the experimental history of the lungs, performed the histological evaluation on coded samples. Three random sections of the right lung from each animal were examined with particular reference to alveolar and interstitial damage defined as cellular inflammatory infiltrates, pulmonary edema, disorganization of lung parenchyma, alveolar rupture, and/or hemorrhage. A semi quantitative morphometric analysis of lung injury was performed in 3 random sections of the right lung from each animal by scoring 0 to 4 (none, light, moderate, severe, very severe) for each of the following parameters: cellular inflammatory infiltrates, edema, disorganization of lung parenchyma, alveolar rupture, and/or hemorrhage, as previously described and validated by our group [17]. A total histological lung injury score was obtained by adding the individual scores in every animal and averaging the total values in each group.

### RNA extraction and reverse transcription

Left lungs were excised, washed with saline, frozen in liquid nitrogen, and stored at -80°C for subsequent RNA extraction. Lungs were homogenized and total lung tissue RNA was purified using TRIreagent (Sigma, Germany) and DNase I digestion (Amersham Biosciences, Essex, United Kingdom) [18]. Five μg of total RNA were subsequently used to synthesize cDNA using the First Strand cDNA synthesis kit (Roche, Switzerland). Expression levels of tumour necrosis factor-alpha (*TNF*-α), interleukin-6 (*IL6*), and *IRAK3 *genes for all samples were determined by using SYBR green I (Molecular Probes, Leiden, The Netherlands) and the iCycler iQ Real-Time detection System (Bio-Rad Laboratories, CA). The β-actin gene was amplified and used as a housekeeping gene. Real-Time amplification reactions were performed using previously published primer pairs [4,19], except for the *IRAK3 *gene whose primers were designed for rat-mouse-human cross-species gene specific amplification (5'-CATCTGTGGTACATGCCAGAAG-3' and 5'-CCAGAGAGAAGAGCTTTGCAG-3'). Relative expression levels were obtained from three serial dilutions of cDNA (each by triplicate) using the ΔΔC_T _method. All fragments were checked for specificity by direct sequencing of both strands with an ABI PRISM 310 Genetic Analyzer using Big Dye Terminator kit v 3.1 (Applied Biosystems, CA).

### Cytokine serum levels

At the end of every experiment, 2 ml of blood was collected from each rat by cardiac puncture and centrifuged for 15 min at 3,000 rpm. Sera were divided into aliquot portions and frozen at -80°C. TNF-α and IL-6 protein concentrations in serum were measured by commercially available immunoassays (Cytoscreen, Biosource International, Camarillo, CA) and performed according to the manufacturer's specifications using an ELx800 NB Universal Microplate Reader (Bio-Tek Instruments, Winooski, Vermont, USA). TNF-α and IL-6 concentrations are expressed as pg/ml. The threshold sensitivity was 8 pg/ml for IL-6 and 4 pg/ml for TNF-α.

### Total protein extraction and Western inmunoblotting

Detection of TLR2, TLR4, IκBα, and IRAK-3 protein expression was performed by Western blotting. Lungs were processed for total protein using ice-cold Nonidet P-40 lysis buffer containing 1% Nonidet P-40, 25 mM Tris-HCl (pH = 7.5), 150 mM sodium chloride, 1 mM EDTA, 5 mM sodium fluoride, 1 mM sodium orthovanadate, 1 mM phenylmethylsulfonyl fluoride plus Protease Inhibitor Cocktail (Roche Molecular Biochemicals, Switzerland) as previously described [20]. Protein concentrations in each experimental condition were determined by the DC Protein Assay (Bio-Rad, CA). Samples were electrophoresed in 10% SDS-PAGE gel, transferred to PVDF membranes, and blocked with 10% skim milk in Tris-buffered saline plus 0.1% Tween 20 (TBS-T). After incubation with TLR2, TLR4, IκBα, IRAK-3 primary antibodies reacting with mouse, rat, and human epitopes (Santa Cruz Biotechnology Inc, Santa Cruz, CA and Abcam^®^, Cambridge, UK), blots were incubated with secondary antibody linked to HRP (Goat Anti-rabbit IgG-HRP; Santa Cruz Biotechnology Inc, Santa Cruz, CA). Bands were visualized using enhanced chemiluminescence (Amersham ECL Western Blotting Detection Reagents, GE Healthcare). For load control, membranes were stripped using Restore Western Blot Stripping Buffer and re-probed with β-actin primary antibody (Cell Signaling Technology) and the same secondary antibody. Densitometric quantification of data was performed using the Scion Image software package.

We used a cell line of human lung fibroblasts IMR-90 (American Type Culture Collection), as positive control for TLR2 protein levels. Cells were grown to sub confluence in Dulbecco's modified Eagle's medium supplemented with 10% FBS, penicillin (100 U/ml) and streptomycin (100 ng/ml) and incubated at 37°C with 5% CO_2_. Total extracts and western blot analysis were performed using the same methods.

### Immunohistochemistry for IRAK-3

Immunohistochemical stains were performed applying a standard avidin-biotin complex (ABC) technique. Fresh frozen sections (5 μm) of rat lung were mounted onto glass slides, fixed in acetone, air dried, and rehydrated in PBS. After blocking endogenous peroxidase activity (10 min in 0,3% hydrogen peroxide), slides were incubated for 1 hour at room temperature with the rabbit polyclonal anti-IRAK-3 antibody (Abcam, Cambridge, UK), then washed in PBS and incubated for 10 min with biotinylated goat anti-rabbit secondary antibody (Santa Cruz Biotechnology Inc, Santa Cruz, CA). Following another washing cycle, slides were incubated for 13 min at room temperature with horseradish peroxidase (HRP)-conjugated streptavidin (Zymed, San Francisco, CA), and for 20 minutes at room temperature with AEC+/substrate Chromogen (Dako, Hamburg, Germany). Finally, sections were rinsed in distilled water, counterstained with hematoxylin, washed in running tap water, and mounted with mounting media (Dako, Hamburg, Germany). Slides were viewed using an Olympus (BX50) microscope and were photographed with an Olympus Camedia digital camera at ×400 magnification.

### Statistical analysis

Statistical analysis was performed with the Fisher exact test and paired and unpaired Student *t*-tests, as appropriate. Comparisons that involved all groups of animals were performed with one-way analysis of variance. If a difference was found, Student *t*-test was applied. Values derived from cytokine gene expression were expressed as group median, normalized by the lowest levels of gene expression in the group, and tested with the Kruskall-Wallis test and the U-Mann Whitney test. Data from ELISA were analyzed by the Student-Newman-Keuls all pairwise multiple range test. Data analysis was performed using SPSS 15.0 (SPSS Inc, Chicago, IL). A value of *p *< 0.05 was considered statistically significant.

## Results

### Outcome and pathophysiologic evaluations

**Figure 1:**
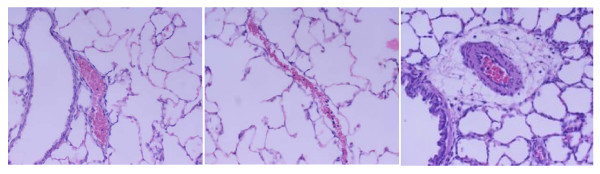
**Representative histopathologic features of lungs from all animal groups**. *Left panel*: normal, unventilated lung; *Middle panel*: low tidal volume (6 mL/kg): lungs did not exhibit significant changes compared to healthy, unventilated lungs. *Right panel*: high tidal volume (20 mL/kg): inflammatory infiltrates and perivascular edema (hematoxylin & eosin staining; original magnification ×200).

All animals survived the 4-hour period of spontaneous breathing or mechanical ventilation at low and high V_T_. Respiratory rate was 90 ± 0.5 cycles/min in the low VT group and 30 ± 0.5 cycles/min in the high VT group. Mean peak airway pressure during the study period was 14 ± 1 and 24 ± 2 cmH_2_O in the low V_T _and high V_T _groups, respectively. Lungs from animals ventilated with high V_T _had acute inflammatory infiltrates and perivascular edema (Figure 1) whereas there were no major histological differences between animals ventilated with low V_T _compared to spontaneously breathing animals. At the end of the 4-h ventilation period, animals ventilated with high-V_T _had higher histological injury scores than low-V_T _animals (6 ± 1 vs. 0.9 ± 0.2, p < 0.0001).

### Pro-inflammatory cytokine gene expression in the lungs and protein serum levels

**Figure 2:**
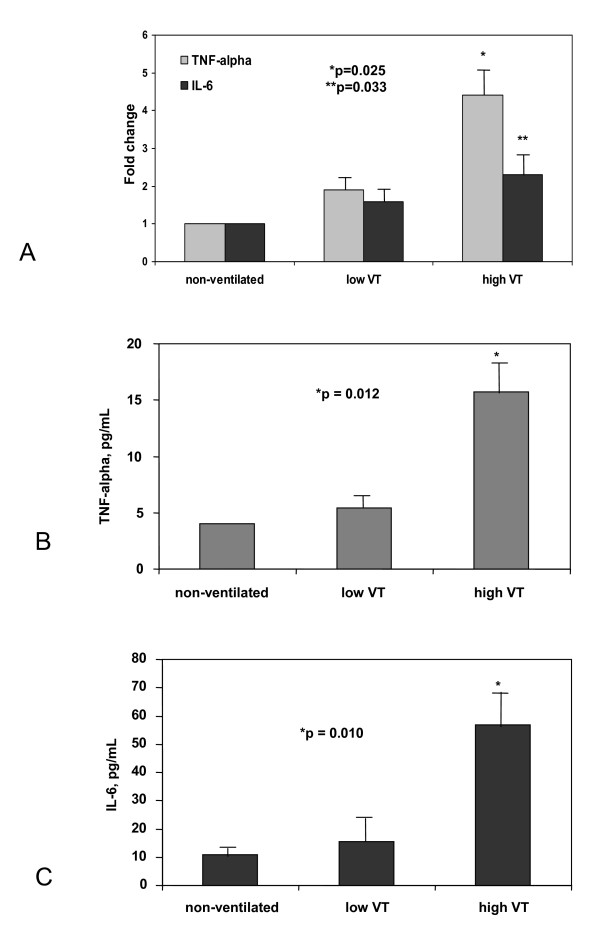
**A) Fold changes of *TNF-α *and *IL6 *mRNA levels in the lungs of healthy rats after 4 hours of spontaneous breathing (non-ventilated), and on mechanical ventilation with 6 ml/kg (low V_T_), 20 ml/kg (high V_T_)**. Bars represent the median of six rats per group. *p = 0.025 when compared to low V_T_; **p = 0.033 when compared to spontaneous breathing animals. **B and C) **Effects of 4 hours of spontaneous breathing in non-ventilated, anesthetized animals and of 4 hours of mechanical ventilation with low V_T _**(B) **and high V_T _(**C**) on systemic protein levels of TNF-α and IL-6. Bars represent the mean values of 6 rats per group.

High V_T _MV up-regulated *TNF-α *gene expression in the lungs of healthy animals (p = 0.025). Although MV with a V_T _of 6 or 20 ml/kg did not significantly change *IL6 *gene expression (p = 0.146), significant differences were found between spontaneous breathing and high V_T _groups (p = 0.033) (Figure 2A). After 4 hours of low V_T _MV, serum concentrations of TNF-α were not significantly different compared to spontaneously breathing animals. Animals ventilated with high V_T _had a significant increase in TNF-α and IL-6 serum levels (p = 0.012 and p = 0.010, respectively) (Figure 2B and 2C).

### Mechanical ventilation induced NF-κB and up-regulated TLR4 but not TLR2

**Figure 3:**
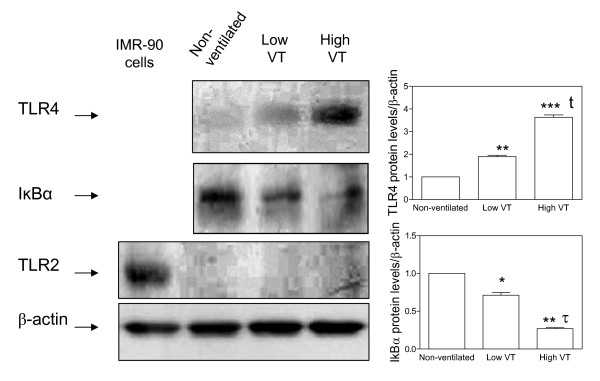
**Effects of V_T _on protein levels in the lungs for TLR2, TLR4, and IκBα, analyzed by Western blotting in animals ventilated with low or high tidal volume for 4 h**. (*) *p *< 0.05 *vs*. non-ventilated animals, (**) *p *< 0.01 *vs*. non-ventilated animals, (***) *p *< 0.001 *vs*. non-ventilated animals, t *p *< 0.001 *vs*. animals ventilated with low VT, τ p < 0.01 vs. animals ventilated with low V_T_. IMR-90 cell line was used as positive control for TLR2 protein levels. Note that these antibodies react with mouse, rat, and human epitopes. Data are reported as mean ± SD and were obtained from 6 animals in each group. V_T _= tidal volume.

As shown in Figure 3, mechanically ventilated animals up-regulated TLR4 but not TLR2 protein levels in the lungs. Furthermore, the highest TLR4 protein levels were found in animals ventilated with high V_T _compared to non-ventilated animals and those ventilated with low V_T _(*p *< 0.001 in both cases). MV induced degradation of IκBα (as a proxy for NF-κB activation) to a greater extent in animals ventilated with a high V_T _compared to non-ventilated animals and animals ventilated with low V_T _(*p *< 0.01) (Figure 3).

### *IRAK3 *gene expression and protein levels in the lungs

**Figure 4:**
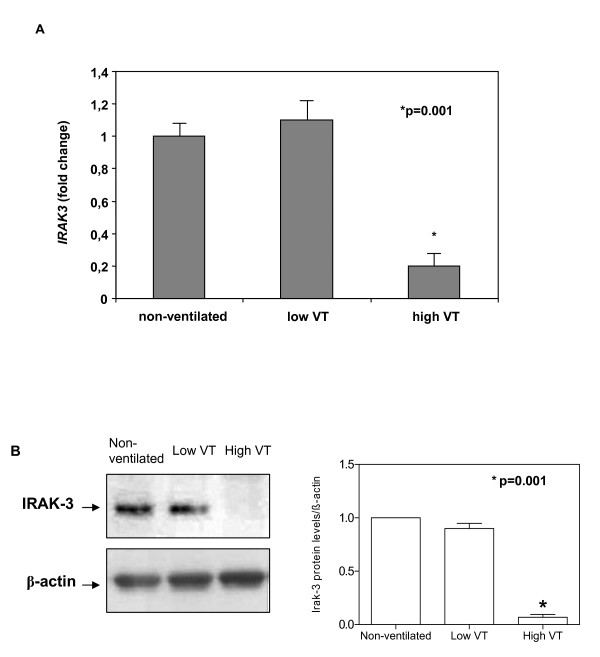
**A) Fold changes in *IRAK3 *gene expression in healthy lungs after 4 hours of spontaneous breathing (non-ventilated) or mechanical ventilation with 6 ml/kg and 20 ml/kg**. Bars represent the median fold-increase compared to non-ventilated animals. (*) p = 0.001 *vs*. non-ventilated animals. **B) Representative blots from individual animals showing changes of IRAK-3 protein levels in lungs after 4 hours of mechanical ventilation with low or high V_T_.** Histograms represent mean densitometric values showing IRAK-3 protein levels from all animals in each group (n = 6 animals per group). Data are reported as means ± SD and were obtained from 6 independent experiments. (*) p = 0.001 *vs*. non-ventilated animals. V_T _= tidal volume.

*IRAK3 *gene expression in the lungs varied depending on the ventilatory strategy: animals from the non-ventilated and low V_T _groups had similar levels of *IRAK3*; however, mechanical ventilation with 20 ml/kg was accompanied by a significant decrease of *IRAK3 *gene expression (p = 0.001) (Figure 4A). Protein levels of IRAK-3 in the lungs were similar in spontaneous breathing animals and in those ventilated with low V_T_. However, IRAK-3 protein levels were markedly reduced after 4 hours of high V_T _mechanical ventilation (*p *= 0.001) (Figure 4B), paralleling gene expression results.

### Immunohistochemical localization of IRAK-3 in the lung

**Figure 5:**
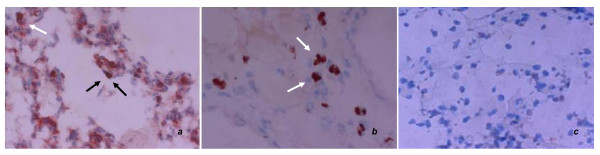
**Immunohistochemichal localization of IRAK-3 in lung tissues of A) spontaneous breathing rats, B) rats ventilated at low (6 ml/kg) tidal volume, and C) rats ventilated at high (20 ml/kg) tidal volume**. *Black *and *white arrows *in A and B point to cytoplasmatic and nuclear staining of epithelial type II cells and interstitial macrophages surrounding the alveolus, respectively. The inflammatory infiltrate with monocytes and lymphocytes and the absence of detectable IRAK-3 in C are due to the effect of high V_T _ventilation. Results are from at least 8 independent experiments. Tissues are counterstained with hematoxylin. *Original magnification *×400.

Lung immunohistochemistry supported the down-regulation of IRAK-3 during high V_T _MV (Figure 5). In particular, positive cytoplasmatic and nuclear staining for IRAK-3 was found in the alveolar lining (epithelial type II cells) and in the interstitial space (monocytes/macrophages) in the lungs of spontaneous breathing animals and those ventilated with low V_T_. However, positive staining for IRAK-3 was minimal in lungs ventilated with high V_T_.

## Discussion

The main findings of this study were the observations that high V_T _MV, in the absence of infection, induced up-regulation of TLR4 and down-regulation of IRAK3 and IκBα protein levels, resulting in an increase of pro-inflammatory cytokines levels in the lungs and in the systemic circulation. These findings suggest that inappropriate MV may represent a stimulus for the immune system similar to that elicited by severe bacterial infections [3,4].

The ability of MV to regulate the innate immune response to high V_T _ventilation is consistent with prior reports documenting an induction of NF-κB [21] and pro-inflammatory cytokine production [2-4]. Gene expression profiles obtained from microarrays across different experimental models of VILI also suggest that the response triggered by alveolar overdistension might mimic an innate immune inflammatory response against pathogens [22]. *In vitro *studies have shown that mechanical stretch is a potent stimulus for growth, differentiation, migration, remodeling, and gene expression from a variety of lung cells including alveolar epithelial cells, endothelial cells, macrophages and fibroblasts [7,8,23-28]. *Ex vivo *studies have demonstrated that injurious ventilatory strategies in both isolated non-perfused rat lungs and isolated perfused mouse lungs cause an increase in the induction and release of inflammatory mediators [2,3,29]. *In vivo*, injurious mechanical ventilation can cause an increase in pulmonary and systemic inflammatory cytokines [4,30]. Tremblay et al [3] ventilated isolated lungs during 2 hours with a V_T _of 15 ml/kg and zero PEEP and found that average peak pressures increased 2.5-fold in the first 30 min (from 9 to 23 cmH_2_O) and 2-fold (from 13 to 28 cmH_2_O) by the end of 2-hour period compared to control lungs. We found that average peak pressures in healthy lungs ventilated with 20 ml/kg increased almost 2-fold (from 14 to 24 cmH_2_O) by the end of 4-hour period when compared to those animals ventilated with 6 ml/kg. Ventilatory strategy also modulates alveolar and plasma levels of pro-inflammatory cytokines in patients with acute lung injury [5,31].

The ability of MV to induce inflammation may be in part explained by its known ability to modulate the induction of NF-κB in response to injurious ventilation alone or in combination with bacterial products [32]. In an isolated perfused mouse lung model, Held et al [21] found that both overinflation of the lung (V_T _of 32 mL/kg) for 150 min and LPS treatment caused activation of NF-κB in lung tissue and resulted in the release of a similar cytokine profile. These experiments were performed during the same period in which IRAK-3 was originally identified by Wesche et al in 1999 [33], and therefore did not explore the possibility that deregulation of genes participating in the endogenous TLR-signaling cascades could be involved in the activation of NF-κB. Increased TLR expression and/or signaling may contribute to the pathophysiology of several important disease states since blunting the up-regulation of *TLR e*xpression with an immunomodulator was correlated with improved outcome [34]. We observed that high V_T _MV for 4 h induced up-regulation of TLR4 (and not TLR2) protein levels, a receptor related to LPS signal transduction and the classical NF-κB pathway [9]. Although our study was performed in rats with healthy lungs, it may be possible that in addition to overdistension by high V_T_, the lack of application of a low level of PEEP (2-5 cmH_2_O) could contribute to ventilator-induced lung damage by causing the opening and closing of lung units (volutrauma and atelectrauma) with every respiratory cycle. Recently, Vaneker et al [16] reported the effects of ventilation for 4 hours with 8 mL/kg V_T_, 4 cmH_2_O of PEEP and 40% oxygen in healthy and knockout TLR2 and TLR4 mice. They found that MV of healthy mice resulted in increased expression of endogenous TLR4 ligands in the bronchoalveolar lavage fluid and enhanced *TLR4 *lung gene expression in lung homogenates that was associated with increased levels of TNF-α and IL-6 in lung and plasma. However, in TLR4 knockout mice, MV did not increase plasma levels of those cytokines. Therefore, their study also suggests that TLRs play a role in the inflammatory response initiated by MV in healthy lungs. Our study is complementary to a study by Moriyama et al [35] who found that animals ventilated with high V_T _(20 ml/kg) for 4 hours had increased expression of receptor CD14 mRNA and protein in the absence of LPS stimulation.

**Figure 6:**
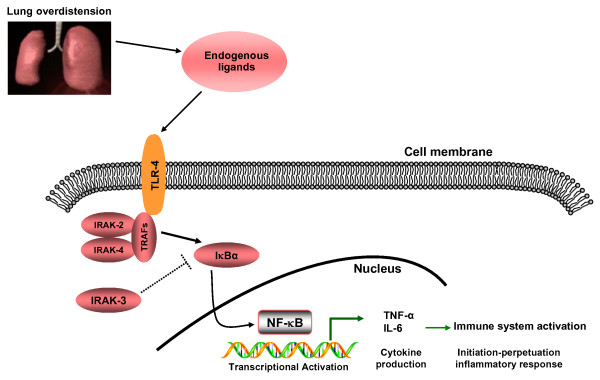
**Proposed TLR/NF-κB signaling pathway activation in a non-infectious, high VT mechanical ventilation experimental model**. Overdistension induced by high VT mechanical ventilation produces endogenous ligands that are able to activate TLR-4 receptors. Subsequent activation of downstream intracellular adapter proteins, enhanced by the down-regulation of *IRAK3 *expression, leads to the degradation of Iκβα and activation of NF-κB, which gives rise to the expression of pro-inflammatory cytokines. Abbreviations: TLR-4: Toll-like receptor-4; IRAK: Interleukin-1 receptor-associated kinase; IκBα: Inhibitory kappa B alpha; NF-κB: Nuclear factor kappa B; IL-6: Interleukin-6; TNF-α: Tumor necrosis factor-alpha. TRAF: Tumor necrosis factor receptor-associated factor; VT: tidal volume.

IRAK-3 is a well described repressor of NF-κB signalling and successful induction of pro-inflammatory signals requires loss of IRAK-3 from the NF-κB pathway [13]. Although IRAK-3 was originally described in monocytes and macrophages [33], and it is primarily present in the peripheral blood leukocytes and monocytic cell lines [36], subsequent studies have reported that it is also expressed in other cell types. Balaci et al [37] found that IRAK3 is highly expressed in alveolar epithelial cells, congruent with our results (see Figure 5). Our findings suggest that MV functions as a modulator of the inflammatory response in the lung via the IRAK-3 immune effects in alveolar macrophages and type II cells. Although totally speculative, we think that this pivotal role of IRAK-3 in preventing excessive activation of NF-κB and subsequent inflammatory response may also be exploited by other cells. In this study, we have shown that, in non-infected animals, MV for 4 h induced IRAK-3 down-regulation, TLR4 up-regulation and that different patterns of MV caused different patterns of IRAK-3 expression. This is congruent with an enhanced NF-κB activation directly caused by IRAK-3 deficiency [38]. Kobayashi et al [13] also showed that macrophages from IRAK-3-deficient mice produced markedly enhanced levels of inflammatory cytokines in response to TLR stimulation. This loss of IRAK-3 protein levels could be responsible for the increased cytokine production. However, the mechanism for loss of IRAK-3 expression remains incompletely understood. Components of the extracellular tissue matrix (including proteoglycan, collagen and elastin) could play a key role in the unremitting inflammation during ventilator-induced lung injury [34,39,40]. Moriondo et al [39] examined the effects of stretching lung tissue during 4 hours of MV at various V_T _with zero PEEP in the lungs of healthy animals and found that significant fragmentation and degradation of the components of the extracellular tissue matrix were observed after ventilating healthy rats with V_T _≥ 16 ml/kg. Jiang et al [41] demonstrated that extracellular matrix fragments isolated from serum of patients with acute lung injury stimulated macrophage chemokine and cytokine production through a TLR-dependent activation of NF-κB. In addition, down-regulation of *IRAK3 *expression by specific small interfering RNAs have been shown to reinstate the production of TNF-α after re-stimulation of macrophages with cell wall components [20]. Likewise, intracellular molecules released into the circulation have been shown to trigger TLR/NF-κB pro-inflammatory pathways [42]. As postulated by our findings, we have designed a speculative schematic figure for a better understanding of the sequence of events after lung overdistension following the application of high-VT ventilation (Figure 6).

Although our data may imply a role for the TLR4/IRAK-3 system in regulating multiple pro-inflammatory cytokines during MV, we acknowledge some limitations to this study. First, we did not explore whether repression of *IRAK3 *expression during high V_T _MV could be reversed by returning to low V_T_. However in patients with ALI, pulmonary and systemic inflammatory responses induced by temporary application of high V_T _can be reversed by reinstitution of lung protective MV [5], at least over the time frame of a few hours. Second, we do not know whether inhibition of TLR4 with blocking antibodies affect the IRAK-3 response. We cannot say that our data fully demonstrate that TLR4 pathway is conclusively involved in increased inflammation associated with the use of high-VT ventilation because the experiments did not examine the effects of disrupting these pathways, as Vaneker et al [16] have shown in TLR4-/- mice. However, Smith et al [43] reported that the blockade of TLR4 receptor reduced pulmonary inflammation induced by MV and LPS. On the other hand, Ringwood et al [36] have found that IRAK-3-/- macrophages exhibit enhanced NF-κB activity and elevated expression of various inflammatory cytokines upon stimulation with several TLR ligands. Third, there is a possibility that the repression of IRAK-3 expression could be unrelated to the activation of TLR4 signaling and could be governed by other molecules capable of regulation of inflammation [44].

## Conclusions

We have documented a differential pattern of TLR4 and IRAK-3 expression and protein levels in the lungs of previously healthy rats following a 4-h period of MV with low or high V_T_. The current study supports an interaction between TLR4 and NF-κB signaling pathway for the over-expression and release of pro-inflammatory cytokines during ventilator-induced lung injury. Our study also suggests that injurious MV may elicit an immune response that is similar to that observed during severe infections. Further studies are needed to fully address these questions.

## Abbreviations

ELISA: enzyme-linked immunosorbent assay; IκBα: inhibitory kappa B alpha; IL-6: interleukin-6; MV: mechanical ventilation; NF-κB: nuclear factor kappa B; PEEP: positive end-expiratory pressure; TLR2: Toll-like receptor-2; TLR-4: Toll-like receptor-4; TNF-α: tumor necrosis factor-alpha; V_T_: tidal volume.

## Competing interests

The authors declare that they have no competing interests.

## Authors' information

Arthur S Slutsky is Adjunct Professor at King Saud University, Riyadh, Saudi Arabia

## Authors' contributions

JV, CF, RK and AS conceived and designed the study. JV obtained funding for the study. JV, NC, MC, FV, LDF, CF, MM performed the experiments. JV, CF, and FV coordinated data collection and data quality. CF, NC, LDF and MM performed statistical analysis. JV, NC, MC, CF, FV, RK, and AS participated in the first draft of the manuscript. All authors participated in the writing process of the manuscript and read and approved the final manuscript.
